# Evaluation of the Heart Function of Swimmers Subjected to Exhaustive Repetitive Endurance Efforts During a 500-km Relay

**DOI:** 10.3389/fphys.2019.00296

**Published:** 2019-03-22

**Authors:** Robert Gajda, Ewa Kowalik, Sławomir Rybka, Ewa Rębowska, Witold Śmigielski, Michał Nowak, Magdalena Kwaśniewska, Piotr Hoffman, Wojciech Drygas

**Affiliations:** ^1^Center for Sports Cardiology, Gajda-Med Medical Center, Pultusk, Poland; ^2^Department of Congenital Heart Diseases, The Cardinal Stefan Wyszyński Institute of Cardiology, Warszawa, Poland; ^3^Internal Diseases Department, Hospital in Śrem, Śrem, Poland; ^4^Department of Preventive Medicine, Medical University of Łódź, Łódź, Poland; ^5^Department of Epidemiology, Cardiovascular Disease Prevention and Health Promotion, The Cardinal Stefan Wyszyński Institute of Cardiology, Warszawa, Poland; ^6^The Unit of Demography and Social Gerontology, University of Łódź, Łódź, Poland

**Keywords:** endurance swimming, echocardiographic assessment, open-water swimming, exhaustive exercise, transthoracic echocardiography, myocardial function

## Abstract

**Aim:** Knowledge of the human body’s ability to adapt to repeated endurance efforts during swimming is limited. We echocardiographically assessed the impact of an exhausting and repetitive swimming effort on cardiac activity.

**Materials:** Fourteen well-trained amateur swimmers (8 female swimmers aged 16–43 years and 6 male swimmers aged 13–67 years old) participated in an ultramarathon relay. Over 5 days, swimmers swam 500 km in the Warta River (in 5-km intervals). Each swimmer swam seven intervals, each within 44:46 to 60:02 min. Objective difficulties included low water temperatures, strong winds, rain, and night conditions.

**Methods:** Transthoracic echocardiography (TTE) was performed three times: at baseline (the day before exertion), at peak effort, and during recovery (48 h after the event). The heart rate (HR) of each swimmer was monitored.

**Results:** Swimmers completed the ultramarathon relay within approximately 91 h. The average HR value at the end of each interval was 91% HRmax. TTE test results showed no significant changes indicative of deterioration of myocardial function at peak effort or after 48 h. Significant increases in left ventricular (LV) ejection fraction, LV fractional shortening (LVFS), LV myocardial systolic velocity, and right ventricular (RV) fractional area changes observed on day 2 after swimming were compared to baseline values and peak effort values. No significant changes in diastolic heart function were observed.

**Conclusion:** Echocardiography assessment indicated that prolonged intense swimming does not affect LV or RV function. Supercompensation of the post-event RV function and increased global LV systolic function demonstrated ventricular interaction after prolonged intense swimming.

## Introduction

Knowledge regarding the adaptations of the human body following long hours of exhaustive swimming is limited (Alexiou et al., 2005; Drygas et al., 2014; Stepien et al., 2017). However, in many countries, including Great Britain and the United States (Nikolaidis et al., 2018), Switzerland, Italy (Rüst et al., 2014b), Canada (Rüst et al., 2014a), Australia, and Poland, long-distance races and swimming marathons, such as the Manhattan Island Marathon Swim (Knechtle et al., 2014a), the Catalina Channel Swim (Knechtle et al., 2015), and the Zurich Lake Marathon Swim (Eichenberger et al., 2013), are becoming increasingly popular (Knechtle et al., 2014b). Every year, many swimmers undertake extremely difficult challenges such as attempting to cross extremely long distances in seas, lakes, and rivers (Khodaee et al., 2016; Valenzano et al., 2016). More than 1800 swimmers, including 571 females from more than 40 countries, successfully completed swimming the English Channel (approximately 32.2 km) prior to 2013 (Knechtle et al., 2014b). Unfortunately, long-distance swimming is associated with high risks to the health of the swimmer. Open-water swimming in seas, lakes, and rivers is particularly associated with distinct threats to health (Nelemans et al., 1994; Castro et al., 2009; Tipton and Bradford, 2014). According to information provided by the media, some swimmers who undertake extremely long, uninterrupted efforts must stop due to extreme fatigue, hypothermia, hyperthermia, or injuries caused by dangerous sea creatures (Keith, 2014; Halliday, 2018; Amazon Swim, 2019; Caldas, 2019). Grünig and co-authors reported 38 cases of swimming-induced pulmonary edema (SIPE) selected from 17 works (Grunig et al., 2017). The literature also describes dozens of sudden deaths and cardiac arrests of triathletes while participating in the swimming part of competitions (Harris et al., 2017). Deaths among high-level competitors during swimming marathons have also been reported (Harris et al., 2017; Smith et al., 2017). In our recently published studies, we presented detailed changes in biochemical and hematological indicators and clotting and fibrinolysis parameters experienced by a 61-year-old swimmer who swam solo for a distance of 120 km over the course of 27 h without leaving the river (Drygas et al., 2014; Stepien et al., 2017).

Although the benefits of systematic training for physiological capabilities and the health status of athletes are indisputable, numerous studies have indicated that multi-hour, exhausting physical effort may cause heart rhythm disturbances, heart attacks, and even sudden death during or after exertion (La Gerche et al., 2012; O’Keefe et al., 2012; Vitiello et al., 2013; Eijsvogels et al., 2016). Determining whether systematic, long-lasting, multi-hour endurance efforts can lead to dysfunction and even permanent damage to the heart is among the most important and most controversial problems in sports medicine. Previous studies involving ultramarathon runners showed that, in some cases, worsening of the heart function was observed by echocardiography at the peak of effort (Spirito et al., 1994). Typically, these changes are related to the right ventricular (RV) function and are transient (Leischik and Spelsberg, 2014; Rimensberger et al., 2014). Several authors have hypothesized that acquired arrhythmogenic RV cardiomyopathy develops as a result of repeated extreme and long-term endurance efforts (La Gerche et al., 2012, 2017; Heidbuchel, 2018). Other authors, citing their own long-term research, have strongly denied this possibility (Knackstedt et al., 2015; Leischik, 2015). Undoubtedly, health status based on age and sex is significant, but the level of training of athletes and the type and duration of the physical effort are also important. In addition, ruling out the potential contribution of illegal pharmacological assistance used in some sports by both professional athletes and leisure sports participants is difficult.

The aim of our study was to evaluate several vital functions during and after exhausting, repetitive endurance swimming of a group of well-trained, but not elite, swimmers. The swimmers included females and males aged 13–67 years who participated in a 500-km ultramarathon swim relay in open water. Our study included several anthropometrical, biochemical, hematological, and hemostatic parameters and evaluated endothelium function and intraocular pressure. We describe data related to the influence of endurance swimming on cardiac function and structure. We hypothesized that repeated exhausting effort, such as long-distance swimming in a relay swimming ultramarathon, may adversely affect cardiac function, as assessed by echocardiography.

## Materials and Methods

Our study involved a group of swimmers who undertook an extraordinary physical effort: swimming 500 km in a relay race in the Warta River in Poland. The event began on July 15, 2016, at 9:00 pm, between kilometers 292 and 297 of the Warta River. The distance was repeated 90.5 times, and the final 48 km ended in Poznań at kilometer 244 of the Warta River on July 17, 2016, at 4:00 pm. Swimmers swam a total distance of approximately 500 km. Fourteen well-trained non-elite swimmers, including eight females 16–43 years old and six males 13–67 years old, participated in the relay. The athletes alternated turns swimming during the relay; each time they started, they jumped into the water from the boat after the previous swimmer finished a 5-km shift. The competitors swam each shift within 44:46 to 60:02 min depending on the time of day, water temperature, and atmospheric conditions (i.e., strong wind, rain, etc.). Only athletes who completed the relay and for whom we possessed echocardiographic data of all three measurements (12 swimmers; 7 females, and 5 males aged 13–67 years) were included in the statistical analysis. One 15-year-old girl was unable to continue the event on the third day due to acute symptoms of airway inflammation. She was examined by experienced physicians and withdrawn from the relay. Notably, she was free of serious health-related symptoms within 2 days. Another participant, a 43-year-old woman, successfully completed the event, but was unable to participate in the final echocardiography examinations, which were performed 48 h after the event, due to professional obligations. The detailed demographic, clinical, and echocardiographic characteristics of the subjects are reported in Table 1. Table 1 also presents basic anthropometric and physiological data characterizing the participants in this swimming relay event. Notably, there was a wide range of ages (13–67 years; mean, 30 ± 15 years). Furthermore, all relay participants had a slim body structure, low fat content, and, in most cases, lower HR (<60/min) at rest. The VO_2_max values obtained during a stress test performed on a moving treadmill indicated that the swimmers were not high-performance athletes with very high aerobic capacity (Bhat and Shaw, 2017).

**Table 1:** Baseline demographic, clinical, and echocardiographic data of the study participants.

Participant	Age (year)/sex	Height (cm)/weight (kg)	BMI (kg/m^2^)	BSA (m^2^)	HR (beats/min)	Systolic blood pressure (mmHg)/diastolic blood pressure (mmHg)	VO_2_ max (mL/kg/min)	LVEDD (cm)/LVESD (cm)	IVS (cm)/PW (cm)	LV EF (%)	LAD (cm)	RVEDD (cm)
1	67/M	177.3/63.5	20.2	1.8	46	140/82	42.2	5.0/3.5	1.0/1.1	66.0	3.7	2.9
2	38/M	181.0/76.0	23.2	2.0	72	120/75	37.5	4.8/3.0	1.0/0.8	66.6	3.2	2.6
3	31/F	171.0/54.5	18.6	1.6	52	105/70	39.0	4.9/3.1	0.9/0.6	66.0	3.3	2.8
4	43/F	166.6/58.2	21.0	1.6	55	100/65	38.9	4.8/3.2	0.8/0.8	63.4	2.9	2.9
5	16/F	158.5/48.0	19.1	1.5	60	115/70	39.8	4.8/3.0	0.8/0.9	67.0	3.0	2.6
6	29/M	180.3/75.0	23.1	1.9	83	120/77	36.6	5.3/3.4	0.9/1.0	65.0	3.1	2.2
7	18/F	170.5/61.0	21.0	1.7	62	75/62	43.1	5.1/3.2	0.8/0.8	68.0	3.4	2.8
8	16/M	158.0/63.0	25.2	1.7	63	147/68	52.2	5.0/3.1	0.9/0.7	67.9	3.0	3.0
9	23/M	163.5/63.0	23.6	1.7	65	110/70	42.4	4.7/3.3	0.8/0.8	60.0	3.1	2.6
10	24/F	176.5/66.6	21.4	1.8	55	130/80	46.5	4.7/2.9	1.0/0.8	66.8	3.3	2.7
11	39/F	162.7/55.0	20.8	1.6	45	145/95	50.5	5.2/3.4	1.0/0.8	63.9	3.5	2.9
12	13/M	157.8/54.5	21.9	1.6	82	125/75	36.7	4.3/3.0	0.9/1.0	58.8	3.1	2.7


*F, female; M, male; BMI, body mass index; BSA, body surface area; HR, heart rate; LVEDD, left ventricular end-diastolic diameter; LVESD, left ventricular end-systolic diameter; IVS, interventricular septum; PW, posterior wall; EF, ejection fraction; LAD, left atrial diameter; RVEDD, right ventricular end-diastolic diameter*.

The organizers of this extraordinary swimming ultramarathon carefully prepared the entire event while considering the physical and psychological preparations of the relay participants and their safety during the many hours of swimming. All participants had been regularly swimming for at least 5 years, usually 2 to 3 times per week for 45 to 60 min each time. Importantly, the swim ultramarathon participants were mostly well-trained, but non-competitive, athletes. During the 6 months preceding their participation in the ultramarathon, swimmers increased both the frequency and duration of their swimming training. Several training sessions were conducted in natural water reservoirs (lakes and rivers) at low air and water temperatures and at night to adequately prepare them for the upcoming ultramarathon. During the last 5 days before the relay event, the swimmers only participated in light training in the form of warm-up exercises to fully rest before the swimming ultramarathon. During the 2 to 3 weeks before the swimming ultramarathon, all swimmers participated in specialized sports medical tests, including detailed interviews and a medical examination, electrocardiogram (ECG), spirometry test, measurement of maximum oxygen consumption (VO_2_max) during a mobile treadmill test, biochemical blood tests, and an eye examination, at the Department of Sports Medicine of the Medical University of Łódź.

### Weather and Living Conditions

Notably, during the relay, all participants lived together as a group in a tent under harsh conditions. The participants slept for a maximum of 3 to 4 h between their swimming shifts; during each shift, they were driven from the base to the boat, where they started and finished their shift. The subjects were instructed to abstain from consuming alcohol and to eat a light meal, such as boiled chicken breasts with steamed vegetables and rice, before the event and during the breaks of the swim relay.

Water and air temperatures during these 5 days drastically changed. The air temperature ranged from 8 to 22°C and the water temperature ranged from 14 to 24°C, depending on the time of day and place of measurement. Additionally, very unfavorable weather conditions in the form of heavy rains and strong winds occurred during the event. Due to the large waves caused by the strong winds, the competitors repeatedly choked on swallowed water, particularly during the night shifts. Therefore, the swimmers’ bodies underwent serious physical and mental stress. Data from other research showed that calorific deficits and low ambient temperatures had significant adverse effects on the body’s functions (Planer et al., 2012; Schnitzler et al., 2018). Weather conditions during the relay are shown in Table 2.

**Table 2:** Weather conditions during the swim relay.

Data	Hour	Water temperature (°C)	Air temperature (°C)	Day	Night
Tuesday, July 12	21:00	24	23	Sun	Clear, starry
Wednesday, July 13	09:00	23.5	21	Sun, overcast	
	21:00	22	20	Overcast, rain, periods of sun	Overcast and starless
Thursday, July 14	09:00	18	16	Heavy rain, overcast	
	21:00	17	14	Heavy rainfall, overcast, gusty winds, waves 20 cm	Dark and starless
Friday, July 15	09:00	16	17	Heavy rain, overcast, periods of sun, windy	
	21:00	16	12	Very cloudy, rain	Windy all night, waves 50 cm
Saturday, July 16	09:00	16	18	Rain, periods of sun, windy	
	21:00	15	10	Showers, overcast	Starless, cold wind
Sunday, July 17	09:00	14	8	Periods of slight sun, overcast, weak rainfall	Dense fog with limited visibility
	16:00	15.5	22	Periods of sun from 2:00 pm, weakening wind	


### Transthoracic Echocardiography

Each participant underwent an echocardiogram three times as follows: the day before the effort, 48 h after the effort at an echocardiographic laboratory, and during peak effort (i.e., after the final exit from the water, on a boat, in a cabin particularly adapted for this purpose). Each test was performed using the same equipment. All studies were performed and interpreted by a single experienced investigator.

Participants underwent standard Doppler echocardiography using an ultrasound imaging system (Digital Portable Color Doppler SonoScape S8 Exp [S8EXP/S9PRO]; SonoScape Medical, Corp., Shenzhen, China) with a 2.5-MHz transducer and Doppler tissue imaging. Echocardiographic variables, including the left ventricular (LV) end-diastolic diameter, LV end-systolic diameter, septal and posterior wall thicknesses, RV end-diastolic diameter, and left atrial diameter (LAD), were recorded in the parasternal view. LV fractional shortening (FS) was calculated as the percentage change in the LV systolic and diastolic dimensions. The LV ejection fraction (LVEF) was calculated using Simpson’s biplane method. The Doppler-derived LV diastolic inflow was recorded in the apical four-chamber view by placing the sample volume at the level of the leaflet tips. In the same view, the RV end-diastolic and end-systolic areas were measured, and the RV fractional area change (FAC) was calculated. Additionally, the tricuspid annular plane systolic excursion (TAPSE) was acquired using the conventional M-mode method at the lateral tricuspid annulus. The LV myocardial tissue Doppler peak systolic (S_m_), early diastolic (E_m_), and late diastolic (A_m_) velocities were measured by placing the sample volume at the septal and lateral angles of the mitral annulus, and the average values of each velocity were calculated. Similarly, pulsed tissue Doppler was used with the sample volume positioned at the lateral corner of the tricuspid annulus to assess its velocity (RV S_m_).

### Heart Rhythm Analyses

Heart rate (HR) measurements during the exercise were performed using Polar V800 HR monitors (POLAR Electro, Kempele, Finland). The results were analyzed by a cardiologist with extensive experience.

### Statistical Analyses

Significant differences between consecutive measurements using echocardiography (first, baseline; second, peak effort; and third, during recovery) were analyzed by an analysis of variance (ANOVA) Friedmann test followed by a Friedmann *post hoc* test. Parameter characteristics are presented as the average value (± standard deviation). All statistical calculations were performed using STATISTICA 12 software. The significance level was set as *p* < 0.05.

### Ethical Issues

The swimming ultramarathon was organized for charity. Both young and very experienced amateur swimmers participated to raise a considerable amount of money for the Oncology, Hematology, and Pediatric Transplantology Clinic in Poznań.

The athletes provided written consent to participate in the swimming ultramarathon, to participate in the aforementioned study, and to have their results published. Parents provided written consent for the teenage swimmers to participate in the swimming ultramarathon and all biomedical tests. Approval was obtained from the Bioethics Committee of the Medical University of Łódź to perform biomedical monitoring of the Warta Marathon participants.

## Results

The average HR value at the end of each shift was 91% HRmax (169 bpm). The average HR during the effort was 157 bpm. This was 84% of the average maximum HR of all athletes. The maximum HR was the value reported by the athletes after previous maximum swimming efforts, such as tests or competitions (in two cases). The HR values of the individual competitors are shown in Table 3.

**Table 3:** Heart rate values of individual competitors obtained during the ultramarathon relay event and the percentages in relation to the maximum heart rate.

				Mean and maximum HR values during the swim relay	

Participant	Age (year)	Sex	Maximum HR of the athlete	Mean HR (% of Maximum HR of the athlete)	maximum HR (% of maximum HR of the athlete)
1	67	M	158	131 (83)	145 (92)
2	38	M	186	148 (80)	165 (89)
3	31	F	197	164 (83)	172 (87)
4	43	F	176	152 (86)	164 (93)
5	16	F	189	177 (94)	197 (104)
6	29	M	195	156 (80)	176 (90)
7	18	F	194	161 (83)	170 (88)
8	16	M	182	155 (85)	160 (88)
9	23	M	197	167 (85)	172 (87)
10	24	F	169	146 (86)	162 (96)
11	39	F	194	158 (81)	165 (85)
12	13	M	200	166 (83)	180 (90)
			Mean 186.4 ± 12,4	157 (84)	169 (91)


Echocardiographic studies showed no obvious LV wall hypertrophy, with a mean septal thickness of 0.88 ± 0.08 cm and a mean posterior wall thickness of 0.84 ± 0.14 cm (Lang et al., 2015). All individuals displayed preserved LV systolic function, with a mean LVEF of 64.9 ± 2.9%, mean LV end-diastolic diameter of 4.87 ± 0.27 cm, and end-systolic diameter of 3.16 ± 0.18 cm.

Regarding the global systolic LV function, compared to measurements obtained at baseline and at peak effort, significant increases in the LVEF and LV shortening fraction were observed during recovery. Similarly, the myocardial systolic peak velocity (Sm) was higher 2 days after swimming (Table 4).

**Table 4:** Left ventricular systolic function.

Echocardiographic parameter	Baseline	Peak effort	Recovery	*p*-Value
LVEDD (cm)	4.87 ± 0.27 [4.88]	4.90 ± 0.59 [4.74]	4.87 ± 0.47 [4.77]	NS
LVESD (cm)	3.16 ± 0.18 [3.13]	3.17 ± 0.39 [3.04]	2.92 ± 0.41 [2.76]	<0.05^∗^
LV EF (%)	64.9% ± 3.0 [66.0]	64.2% ± 5.1 [64.2]	71.6% ± 3.5 [72.3]	<0.05^∗^
LV SF (%)	36.3% ± 2.1 [36.9]	35.2% ± 3.9 [35.3]	41.1% ± 2.9 [41.7]	<0.05^∗^
Myocardial systolic velocity, S_m_ (cm/s)	13.17 ± 1.71 [13.2]	13.13 ± 1.48 [13.3]	14.64 ± 1.27 [14.4]	<0.01^∗^


*Mean ± SD (median value).*

*^∗^Recovery vs. peak effort and recovery vs. baseline.*

*LVEDD, left ventricular end-diastolic diameter; LVESD, left ventricular end-systolic diameter; EF, ejection fraction; SF, shortening fraction.*

Left ventricular diastolic function, as assessed by the standard transmitral flow, was not affected by the extreme exercise (Table 5). Similarly, compared to the baseline assessment results, the left atrial diameter, early and late myocardial peak velocities, and E_m_/A_m_ and E/E_m_ ratios did not change during or after swimming.

**Table 5:** Left ventricular diastolic function.

Echocardiographic parameter	Baseline	Peak effort	Recovery	*p*-Value
Mitral peak E velocity	103.0 ± 18.3 [102.5]	91.2 ± 13.7 [88.8]	104.6 ± 25.0 [10.1.3]	NS
Mitral peak A velocity	64.4 ± 13.5 [62.4]	62.6 ± 10.3 [59.7]	68.5 ± 11.2 [72.0]	NS
Mitral peak E/A ratio	1.64 ± 0.32 [1.60]	1.49 ± 0.31 [1.43]	1.56 ± 0.42 [1.54]	NS
Myocardial early diastolic velocity, E_m_ (cm/s)	17.2 ± 2.8 [17.2]	16.6 ± 3.9 [17.3]	16.8 ± 4.3 [18.0]	NS
Myocardial late diastolic velocity, A_m_ (cm/s)	9.72 ± 2.18 [9.68]	10.44 ± 1.85 [10.64]	9.62 ± 2.98 [8.50]	NS
E_m_/A_m_ ratio	1.86 ± 0.51 [1.99]	1.67 ± 0.60 [1.61]	1.93 ± 0.73 [2.05]	NS
E/E_m_ ratio	6.08 ± 1.23 [5.91]	5.68 ± 1.04 [5.43]	6.46 ± 1.42 [6.25]	NS
LAD (cm)	3.21 ± 0.22 [3.12]	3.25 ± 0.25 [3.27]	3.22 ± 0.27 [3.14]	NS


*Mean ± SD (median value).*

*LAD, left atrial diameter.*

The RV diameters and non-geometric parameters describing the RV function (TAPSE and S_m_) did not significantly differ between consecutive measurements (Table 6). Moreover, compared to the baseline measurement, an increase in the RV FAC was observed 48 h after exercise.

**Table 6:** Right ventricular systolic function.

Echocardiographic parameter	Baseline	Peak effort	Recovery	*p*-Value
RVEDD (cm)	2.71 ± 0.20 [2.74]	2.63 ± 0.39 [2.56]	2.64 ± 0.27 [2.61]	NS
RV myocardial systolic velocity, S_m_ (cm/s)	18.4 ± 3.9 [17.0]	16.9 ± 2.7 [16.7]	18.24 ± 3.4 [16.9]	NS
TAPSE (mm)	24.3 ± 2.8 [24.2]	24.3 ± 3.3 [23.7]	25.6 ± 4.5 [25.0]	NS
FAC (%)	51.5% ± 3.4 [51.3]	56.6% ± 7.4 [58.4]	58.6% ± 6.9 [57.8]	<0.05^∗^


*Mean ± SD (median value).*

*^∗^Recovery vs. baseline.*

*RVEDD, right ventricular end-diastolic diameter; TAPSE, tricuspid annular plane systolic excursion; FAC, fractional area change.*

While separately analyzing the individual echocardiographic data of all ultramarathon participants, we did not detect any changes that could be interpreted as signs of cardiac “fatigue,” dysfunction, or any other cardiac pathology. Full echocardiographic data of the study participants are presented in Appendix 1.

## Discussion

The increasing popularity of endurance and ultra-endurance swimming in many countries is an interesting and intriguing phenomenon (Knechtle et al., 2014b). Unfortunately, despite many excellent studies analyzing factors related to performance, speed, and energy, information regarding the adaptation of cardiac function to endurance or ultra-endurance swimming are scarce (Costa et al., 2015). Moreover, we were unable to find any studies related to repeated exhaustive endurance swimming in open water.

During our study, 14 competitors participated in an ultramarathon swim relay event comprising a distance of 500 km. Three echocardiographic examinations were performed for seven females and five males who completed the ultramarathon and participated in all three tests. Each competitor swam approximately 35 km. Each competitor was assumed to have considered the shift “very intense training.” The average HR value at the end of each shift was 91% HRmax. Some competitors even reached 95 to 104% of their maximum HR (considered maximum until the event) during individual sections of the swimming ultramarathon. Based on these values, we determined whether the intensity of the effort was high, very high, or maximum (Kozłowski and Nazar, 1995) (Table 7). The HR values of the athletes during swimming were considered accurately measured. The records of the HR monitors that we analyzed indicated no unexpected fluctuations in values that could indicate the occurrence of artifacts. In such situations, we can assume that HR monitors used by athletes correctly indicate both the average and maximum HR values (Gajda et al., 2018).

**Table 7:** Division of the intensity of physical effort depending on the achieved heart rate.

	Intensity					
	
	Very low	Low	Medium	High	Very high	Maximum
%HRmax	<35	35–59	60–79	80–89	>90	100


*Kozłowski S, Nazar K. Introduction to clinical physiology. PZWL 1995.*

Swimmers completed the ultramarathon relay within approximately 91 h. Considering the length of the swimming sections (5 km), the duration of the effort, the number of repeated trials (seven times), the special conditions (the need to swim at night and in low water and air temperatures), and the HR values reached during each shift, the relay was considered exhaustive or extremely difficult (Kozłowski and Nazar, 1995).

The data from our study strongly demonstrate that intense prolonged repeated bouts of swimming in open water in difficult environmental conditions did not negatively influence the heart function.

Echocardiography assessment indicated that prolonged intense swimming does not affect LV or RV function.

In the group of swimmers included in this study, atrial enlargement or deterioration of the RV or LV systolic function was not observed during the echocardiographic evaluations of the heart.

Transthoracic echocardiography (TTE) test results showed no significant changes indicative of deterioration of myocardial function at peak effort or after 48 h. Regarding the systolic function of both chambers, compared to the baseline values and the values at peak effort, significant increases in the EF and FS of the left ventricle, myocardial LV systolic velocity, and RV FAC were observed on day 2 after completing swimming. No significant changes in the diastolic function of the heart were observed.

We did not observe a significant decrease in the echocardiographic parameters when assessing RV function during the post-swimming examination. TAPSE and RV systolic velocity illustrating the function of RV longitudinal fibers did not change; moreover, compared to assessments made at baseline and at peak effort, the RV FAC significantly increased during recovery. A possible explanation for this phenomenon might be that the FAC describes not only longitudinal but also oblique and circular myofibers that appear to enhance its function to address the exercise-induced increased contractility demand. Therefore, our study showed that the right ventricle copes with the increase in load during swimming even if the intense exercise is maintained for a long duration and is repeated during a short period. The supercompensation of the post-event RV function and the increase in the global LV systolic function demonstrated the ventricular interaction after high-intensity swimming.

Moreover, while analyzing separately the individual echocardiographic data of al 500-km relay participants, we did not detect any changes that could be interpreted as signs of cardiac “fatigue” dysfunction, or any other cardiac pathology.

The observed changes did not suggest that long-distance swimming caused changes in the echocardiographic parameters of the tested athletes, thus demonstrating no harmful effect on the hearts of athletes.

Studies investigating changes in LV function following prolonged intense exercise have yielded inconsistent data, and studies involving swimmers are scarce (Douglas et al., 1986). Cahill et al. (1979) measured LV dimensions of 14 athletes, including 7 international swimmers, before and immediately after submaximal exercise. They noted significantly larger left ventricular muscle mass and end-diastolic dimensions in swimmers as compared to controls and decrease in LV end-systolic dimension both in swimmers and controls after exercise. According to our results, the echocardiography-derived global LV function was not only unaffected during intense swimming but also significantly increased during recovery. Santoro et al. (2016), who studied competitive water polo players, observed a significant increase in the LVEF immediately after maximal exercise. As mentioned, the different types of exercise might explain why our observations were not made immediately after the effort, but later during recovery. In contrast to our results, Alexiou et al. (2005) observed a reduction in LVFS and LVEF after exhaustive open-water swimming. Studies using Doppler tissue imaging to investigate the LV systolic function of swimmers are lacking. Similar findings of enhanced LV myocardial systolic velocity after racing were observed by Vitiello et al. (2013) when investigating a small group of runners who participated in an extreme mountain ultra-marathon. However, due to the differences in the study groups, environmental conditions, and types of exercise analyzed in the discussed works and our study, it is challenging to make comparisons and draw conclusions.

Regarding the Doppler-derived diastolic parameters, no significant changes were observed in the LV filling pattern in this study. A previous study showed that after ultra-endurance exercise, the peak early transmitral filling velocity significantly decreased (Hassan et al., 2006). In contrast, full-body immersion led to the opposite diastolic filling changes (increase in early velocity) (Marabotti et al., 2013). Furthermore, Santoro et al. (2016) observed a significant increase in both early and late diastolic transmitral velocities immediately after maximal exercise in competitive water polo players. Notably, our observations were obtained under unique exercise conditions (i.e., prolonged intense exercise in the water).

Previous studies have revealed that intensive endurance exercise may lead to RV dysfunction, and that the degree of RV functional impairment depends on the duration of the exercise (La Gerche et al., 2012; Heidbuchel, 2018).

Numerous studies have shown that certain cardiac arrhythmias and their preceding morphological and functional changes in the heart are more common in high-performance athletes than in their inactive peers. These arrhythmias include paroxysmal atrial fibrillation and atrial flutter (Castro et al., 2009), and atrial overload enlargement and/or fibrosis resulting from ischemic damage during extreme long-term effort are considered the cause (Everett et al., 2011; Nielsen et al., 2013). However, no works have specifically found an increased risk of AF or RV arrhythmias specifically in swimmers (Guasch and Mont, 2017). The idea that arrhythmogenic RV cardiomyopathy develops in endurance athletes is controversial (La Gerche et al., 2012, 2017). Numerous studies have shown that immediately after intense, long-lasting physical effort, a significant increase in pressure occurs in the right ventricle that recedes during rest (La Gerche et al., 2014). Transient worsening of RV function in the form of deterioration of the EF and other changes in the right ventricle parameters, both morphological and functional, were also observed (La Gerche et al., 2011). In general, the more prolonged the effort, the more intense the cardiac changes (La Gerche et al., 2011, 2012; Sanz-de la Garza et al., 2017). According to several works, repeated dysfunction of the right ventricle after repeated extreme exertions may eventually lead to acquired arrhythmogenic RV cardiomyopathy (La Gerche et al., 2014; Heidbuchel, 2018).

It should be noted that, in our study, the effort was performed during approximately 45- to 60-min sessions. The resting time between sessions may have allowed partial RV recovery (and possibly LV recovery). In practice, the time from leaving the water by the swimmer until the next shift was 9 h. Accordingly, a repetitive exercise pattern might yield a different cardiac adaptation than swimming 25 km consecutively (Alexiou et al., 2005), swimming a marathon, swimming a triathlon (La Gerche et al., 2012), or swimming for 24 consecutive hours (Drygas et al., 2014). However, there was no reason to assume that a 9-h break between 45- and 60-min efforts repeated seven times was enough to fully rest. Moreover, we instructed our swimmers to perform only very light workouts during the last 5 days before the ultramarathon. Thus, the baseline echocardiographic examinations on the day before the ultramarathon relay were performed for the “rested” heart.

The main strengths of our study were the unique exercise protocol and comprehensive monitoring of various important vital functions during and after the ultramarathon. Each athlete performed several intensive swimming endurance exercise sessions in open water during the day and night under difficult environmental conditions. Moreover, other than two very experienced master athletes who were champion endurance swimmers, most swimmers had not been previously involved in competitive swimming training, although all had experience with long-distance swimming. Despite the age groups used in other studies, we included both females and males of various ages in our study. Echocardiography was performed by the same cardiologist with extensive experience with similar studies. A member of our study team was constantly with the swimmers to monitor the necessary physiological parameters and report potential adverse effects. The biochemical, hematological, anthropometric characteristics, intraocular pressure, and endothelium function analyses are currently being prepared by our research group for publication in another article.

The main limitation of this study was that monitoring of the cardiac, hematological, and biochemical adaptations to prolonged repetitive swimming was performed for a relatively small group of athletes. As previously mentioned, we obtained echocardiography data of 12 males and females. The echocardiographic analysis was limited to standard two-dimensional, Doppler, and tissue Doppler studies. Regional myocardial deformation was not performed. We analyzed a heterogeneous group of swimmers that included adolescents (13- to 16-year-old participants) and adults (males and females). However, cardiac adaptations and remodeling may significantly change in adolescents and between sexes (Finocchiaro et al., 2017). The effort was performed during seven 45- to 60-min sessions. Resting time between sessions may have allowed partial recovery of the right ventricle (and possibly the left ventricle). Despite these limitations, our study was the first devoted to morphological and functional cardiac changes following repeated exhaustive endurance swimming in open water. Therefore, although our observations were limited to a relatively small group of swimmers, the results of our study are relevant to a large group of professional athletes involved in endurance swimming. The data from our study are also interesting in the context of whether prolonged endurance exercise may cause transitory or even permanent pathological changes in the function or structure of the right or left ventricle (all data supporting this study are provided as supplementary information in Appendix 1).

In summary, the results of this study indicated that prolonged intense swimming does not affect LV and RV function, as assessed by echocardiography.

## Conclusion

Echocardiography assessment indicated that prolonged intense swimming does not affect LV and RV function. Supercompensation of the post-event RV function and the increase in the global LV systolic function demonstrated ventricular interaction after prolonged intense swimming.

## Data Availability

All datasets generated for this study are include in the manuscript and/or the Supplementary Files.

## Ethics Statement

This study was carried out in accordance with the recommendations of Resolution No. RNN/230/16/KE of July 12, 2016, Bioethics Commission at the Medical University of Łódź with written informed consent from all subjects. All subjects gave written informed consent in accordance with the Declaration of Helsinki. The protocol was approved by the Bioethics Commission at the Medical University of Łódź.

## Author Contributions

RG and WD conceived the study. WD, MK, ER, and MN designed the study details and supervised the data collection. SR, ER, RG, MK, MN, and WD contributed significantly to data collection. RG, EK, PH, and WD participated in writing the paper and checking the draft for errors. WŚ was the statistician and was involved in designing the study. RG, WD, and EK written by the final version of the manuscript. All authors read and accepted the final version of the manuscript.

## Conflict of Interest Statement

The authors declare that the research was conducted in the absence of any commercial or financial relationships that could be construed as a potential conflict of interest.
