# Supplementary material for: Evaluation of the Heart Function of Swimmers Subjected to Exhaustive Repetitive Endurance Efforts During a 500-km Relay
Source: Front Physiol. 2019 Mar 22;10:296. doi: 10.3389/fphys.2019.00296 (PMC6438895; doi:10.3389/fphys.2019.00296)
Supplement: Supplementary file 1 [file Data_Sheet_1.pdf]

Appendix 1. Full echocardiographic data of the study participants

**Participant**	1	2	3	4	5	6	7	8	9	10	11	12
**Sex**	Male	Male	Female	Female	Female	Male	Female	Male	Male	Female	Female	Male
**Age**	67	38	31	43	16	29	18	16	23	24	39	13
**Echocardiographic parameters: Baseline**												
**LAD (cm)**	3.66	3.17	3.25	2.93	3.04	3.05	3.44	3.00	3.07	3.34	3.45	3.07
**LVEDD (cm)**	4.97	4.75	4.92	4.84	4.80	5.31	5.09	5.00	4.67	4.65	5.15	4.30
**LVESD (cm)**	3.50	3.00	3.10	3.17	3.00	3.40	3.15	3.11	3.26	2.94	3.35	2.98
**RVEDD (cm)**	2.85	2.58	2.78	2.86	2.61	2.23	2.76	3.00	2.60	2.71	2.85	2.69
**Mitral peak E velocity (cm/s)**	105.00	106.00	93.40	119.00	124.00	86.20	102.00	138.00	90.30	103.00	68.20	101.24
**Mitral peak A velocity (cm/s)**	73.44	60.60	62.00	92.00	74.00	39.90	64.10	62.70	55.80	54.00	56.00	78.43
**Mitral peak E velocity/mitral peak A velocity**	1.43	1.75	1.51	1.29	1.68	2.16	1.59	2.20	1.62	1.91	1.22	1.29
**LV EF%**	66.00	66.60	66.00	63.40	67.00	65.00	68.00	67.90	60.00	66.79	63.86	58.82
**LV FS%**	36.00	36.85	36.95	34.49	37.40	35.90	38.00	37.98	39.30	36.90	34.95	31.00
**Medial mitral myocardial systolic velocity, S_m (cm/s)**	9.67	14.26	11.30	12.00	11.40	11.30	12.20	12.60	10.00	11.59	10.20	10.20
**Medial mitral myocardial early diastolic velocity, E_m (cm/s)**	11.00	15.15	12.25	13.80	15.20	12.20	12.80	17.50	16.00	12.25	14.00	16.40
**Medial mitral myocardial late**	8.90	11.14	10.92	14.26	10.00	8.40	7.60	8.32	8.47	7.35	9.30	9.30

**diastolic velocity, A_m (cm/s)**												
**Lateral mitral myocardial systolic velocity, S_m (cm/s)**	15.70	16.26	13.59	17.38	18.20	12.40	18.40	14.85	12.20	13.15	9.80	17.38
**Lateral mitral myocardial early velocity, E_m (cm/s)**	11.60	20.00	20.00	22.95	23.00	21.60	24.20	24.70	17.60	18.27	14.70	25.60
**Lateral mitral myocardial late diastolic velocity, A_m (cm/s)**	20.40	8.86	14.26	7.35	9.40	6.00	8.40	7.87	8.20	7.80	10.00	10.69
**Mean mitral myocardial systolic velocity, S_m (cm/s)**	12.69	15.26	12.45	14.69	14.80	11.85	15.30	13.73	11.10	12.37	10.00	13.79
**Mean mitral myocardial early velocity, E_m (cm/s)**	11.30	17.58	16.13	18.38	19.10	16.90	18.50	21.10	16.80	15.26	14.35	21.00
**Mean mitral myocardial late diastolic velocity, A_m (cm/s)**	14.65	10.00	12.59	10.81	9.70	7.20	8.00	8.10	8.34	7.58	9.65	10.00
**Mean E_m/Mean A_m ratio**	0.77	1.76	1.28	1.70	1.97	2.35	2.31	2.61	2.02	2.01	1.49	2.10
**Myocardial early diastolic velocity/Mean myocardial early diastolic velocity ($E/E_{m)}$ ratio**	9.29	6.03	5.79	6.48	6.49	5.10	5.51	6.54	5.38	6.75	4.75	4.82
**RV FAC (%)**	51.20	54.00	48.00	47.00	60.00	51.30	50.00	50.00	52.20	53.78	52.00	49.00
**RV myocardial systolic velocity, S_m (cm/s)**	26.00	23.00	16.90	16.49	17.20	12.90	16.00	16.65	24.00	19.38	17.00	15.60
**TAPSE (mm)**	23.70	29.00	20.30	24.84	22.35	24.70	25.00	23.00	20.37	26.00	29.00	23.71

Echocardiographic parameters: Peak effort

**LAD (cm)**	3.70	3.44	2.86	3.25	2.80	3.36	3.36	3.28	3.41	3.11	3.14	3.23
**LVEDD (cm)**	6.20	4.32	4.81	4.45	4.60	5.57	5.57	4.88	4.67	4.30	4.97	4.42
**LVESD (cm)**	4.00	3.06	3.37	2.79	3.20	3.59	3.59	2.85	2.85	2.85	3.02	2.84
**RVEDD (cm)**	3.30	2.90	2.70	3.10	2.28	2.30	2.00	2.42	2.99	2.40	2.46	2.65
**Mitral peak E velocity (cm/s)**	96.00	86.00	101.00	104.80	89.12	81.99	86.00	112.00	81.99	88.41	61.31	105.50
**Mitral peak A velocity (cm/s)**	69.00	64.00	69.87	77.70	54.19	50.60	61.00	58.00	58.46	49.90	81.99	56.30
**Mitral peak E velocity/mitral peak A velocity**	1.39	1.34	1.45	1.35	1.64	1.62	1.41	1.93	1.40	1.77	0.75	1.87
**LV EF%**	64.00	56.28	57.00	67.50	57.95	64.00	64.40	72.00	69.52	62.80	69.40	65.62
**LVFS%**	35.00	29.18	29.94	37.39	30.46	35.00	35.60	41.50	39.00	33.70	39.20	35.85
**Medial mitral myocardial systolic velocity, S_m (cm/s)**	11.30	13.00	8.24	12.48	11.59	8.90	9.36	12.04	10.03	9.58	11.59	8.69
**Medial mitral myocardial early diastolic velocity, E_m (cm/s)**	10.69	13.00	11.36	14.00	13.15	15.37	13.59	15.60	11.36	13.81	7.58	15.15
**Medial mitral myocardial late diastolic velocity, A_m (cm/s)**	12.48	10.00	10.25	14.93	9.13	9.13	8.00	10.00	10.25	5.57	10.00	8.00
**Lateral mitral myocardial systolic velocity, S_m (cm/s)**	16.00	16.90	14.48	14.48	18.00	17.16	18.49	18.00	16.00	14.26	12.48	12.00
**Lateral mitral myocardial early velocity, E_m (cm/s)**	14.70	17.00	18.49	21.80	24.20	19.38	24.51	29.80	16.70	20.72	9.13	26.90

**Lateral mitral myocardial late diastolic velocity, A_m (cm/s)**	9.30	16.90	10.92	9.36	12.25	10.70	9.58	9.14	12.26	8.69	15.37	8.47
**Mean mitral myocardial systolic velocity, S_m (cm/s)**	13.65	14.95	11.36	13.48	14.80	13.03	13.93	15.02	13.02	11.92	12.04	10.35
**Mean mitral myocardial early velocity, E_m (cm/s)**	12.70	15.00	14.93	17.90	18.68	17.38	19.05	22.70	14.03	17.27	8.36	21.03
**Mean mitral myocardial late diastolic velocity, A_m (cm/s)**	10.89	13.45	10.59	12.15	10.69	9.91	8.79	9.57	11.26	7.13	12.69	8.24
**Mean E_m/Mean A_m ratio**	1.17	1.12	1.41	1.47	1.75	1.75	2.17	2.37	1.25	2.42	0.66	2.55
**Myocardial early diastolic velocity/mean myocardial early diastolic velocity ($E/E_{m)}$ ratio**	7.56	5.73	6.77	5.85	4.77	4.72	4.51	4.93	5.84	5.12	7.34	5.02
**RV FAC (%)**	58.20	60.00	50.80	58.60	63.00	65.70	63.00	56.00	60.00	47.13	57.20	40.00
**RV myocardial systolic velocity, S_m (cm/s)**	20.27	19.60	21.10	16.71	17.16	11.81	16.70	18.70	14.70	15.15	16.00	14.93
**TAPSE (mm)**	30.28	30.00	26.50	24.80	22.40	23.39	24.00	21.70	20.79	20.90	25.70	20.79
**Echocardiographic parameters: Recovery**												
**LAD (cm)**	3.67	3.17	2.97	2.88	3.13	3.02	3.69	3.43	3.12	3.14	3.42	2.97
**LVEDD (cm)**	5.27	4.90	4.76	4.47	4.51	5.64	5.80	4.85	4.50	4.38	4.78	4.52
**LVESD (cm)**	3.46	2.93	2.70	2.78	2.66	3.90	3.23	2.69	2.73	2.49	2.86	2.57
**RVEDD (cm)**	3.39	2.60	2.62	2.53	2.46	2.64	2.33	2.57	2.69	2.82	2.61	2.44

**Mitral peak E velocity (cm/s)**	93.40	100.00	104.00	134.70	108.00	102.60	78.43	151.00	94.10	131.00	58.46	99.80
**Mitral peak A velocity (cm/s)**	78.43	73.00	84.80	79.14	64.17	61.31	57.04	75.50	47.00	57.00	72.72	71.30
**Mitral peak E velocity/mitral peak A velocity**	1.19	1.37	1.23	1.70	1.68	1.67	1.38	2.00	2.00	2.30	0.80	1.40
**LV EF%**	63.00	70.00	74.00	68.00	72.00	72.66	75.00	75.00	70.00	74.00	70.80	74.20
**LVFS%**	34.43	40.00	43.00	37.70	41.16	42.28	44.00	44.40	39.30	43.20	40.20	43.00
**Medial mitral myocardial systolic velocity, S_m (cm/s)**	13.80	13.80	14.00	12.70	10.25	9.69	11.36	12.25	12.90	11.36	9.58	12.25
**Medial mitral myocardial early diastolic velocity, E_m (cm/s)**	11.14	13.59	14.40	16.40	15.80	10.00	11.59	18.00	12.48	14.00	5.57	12.90
**Medial mitral myocardial late diastolic velocity, A_m (cm/s)**	13.80	11.81	9.58	11.59	8.69	6.60	6.68	9.36	8.69	6.68	9.36	8.00
**Lateral mitral myocardial systolic velocity, S_m (cm/s)**	16.70	19.80	18.72	17.80	18.00	18.00	19.60	16.26	15.60	14.70	15.30	16.70
**Lateral mitral myocardial early velocity, E_m (cm/s)**	9.36	19.60	22.06	21.60	24.95	22.00	26.74	25.40	18.40	21.39	8.47	27.40
**Lateral mitral myocardial late diastolic velocity, A_m (cm/s)**	21.17	9.80	11.59	8.24	8.47	9.69	7.58	7.35	5.79	7.80	15.15	7.35
**Mean mitral myocardial systolic velocity, S_m (cm/s)**	15.25	16.80	16.36	15.25	14.13	13.85	15.48	14.26	14.25	13.03	12.44	14.48
**Mean mitral myocardial early velocity, E_m (cm/s)**	10.25	16.60	18.23	19.00	20.38	16.00	19.17	21.70	15.44	17.70	7.02	20.15

**Mean mitral myocardial late diastolic velocity, A_m (cm/s)**	17.49	10.81	10.59	9.92	8.58	8.15	7.13	8.36	7.24	7.24	12.26	7.68
**Mean E_m/Mean A_m ratio**	0.59	1.54	1.72	1.92	2.37	1.96	2.69	2.60	2.13	2.44	0.57	2.63
**Myocardial early diastolic velocity/mean myocardial early diastolic velocity ($E/E_{m)}$ ratio**	9.11	6.03	5.70	7.09	5.30	6.41	4.09	6.96	6.09	7.40	8.33	4.95
**RV FAC (%)**	70.00	54.00	67.00	59.50	56.90	51.70	52.60	60.70	53.70	49.90	69.00	58.70
**RV myocardial systolic velocity, S_m (cm/s)**	20.00	24.70	22.06	21.83	16.71	14.48	16.71	19.60	16.49	17.16	14.00	15.15
**TAPSE (mm)**	34.80	28.60	23.45	25.29	24.68	24.90	21.29	25.16	21.30	31.18	28.00	18.72
